# Is either anosmia or constipation associated with cognitive dysfunction in Parkinson’s disease?

**DOI:** 10.1371/journal.pone.0252451

**Published:** 2021-06-04

**Authors:** Ming-Zhi Sheng, Ting-Chun Fang, Yi-Huei Chen, Ming-Hong Chang, Chun-Pai Yang, Ching-Heng Lin

**Affiliations:** 1 Neurological Institute, Taichung Veterans General Hospital, Taichung, Taiwan; 2 Department of Medical Research, Taichung Veterans General Hospital, Taichung, Taiwan; 3 Department of Neurology, School of Medicine, National Yang-Ming University, Taipei, Taiwan; 4 Department of Neurology, Kuang Tien General Hospital, Taichung, Taiwan; Philadelphia VA Medical Center, UNITED STATES

## Abstract

**Objective:**

To clarify the association of anosmia or constipation with cognitive dysfunction and disease severity in patients with Parkinson’s disease (PD).

**Methods:**

Newly diagnosed patients with PD (less than 5 years) without a clinical diagnosis of dementia were included from February 2017 to August 2018. The subjects were further divided into subgroups based on whether anosmia occurred and the grade of constipation. The severity of PD motor symptoms was rated using the Movement Disorder Society-Sponsored Revision of the Unified Parkinson’s Disease Rating Scale (MDS-UPDRS), and cognitive functions were evaluated by Montreal Cognitive Assessment (MoCA). Statistical analyses including *t*-tests, chi-square tests, multiple linear regression, and binary logistic regression were used to determine statistical significance.

**Results:**

A total of 107 newly diagnosed PD patients were included in this study. The MoCA score was significantly lower in the anosmia group (*p* < 0.001). Constipation was associated with impaired olfaction in a post-hoc test. The correlation coefficient between MoCA and UPSIT score was 0.41 (*p* < 0.001). Total anosmia and age were associated with cognitive dysfunction (MoCA < 26) (odds ratio, 2.63, *p* = 0.003; 1.10, *p* < 0.001, respectively). The anosmia group had a higher MDS-UPDRS part 3 score with β coefficient of 7.30 (*p* = 0.02). Furthermore, grade 3 constipation was associated with a higher MDS-UPDRS total score with β coefficient of 14.88 (*p* = 0.02).

**Conclusions:**

Anosmia but not constipation was associated with cognitive impairment in PD patients. Nevertheless, severe constipation was associated with impaired olfaction and PD disease severity. We suggest that the propagation of α-synuclein from the olfactory route is distinct from the enteric nervous system, but the intercommunication between these two routes is complex.

## Introduction

Parkinson’s disease (PD) is a multisystem degenerative disorder with both motor and non-motor manifestations. Olfactory dysfunction and constipation are among the earliest non-motor symptoms in patients with PD. In cross-sectional studies, up to 90% of PD patients are affected by some degree of hyposmia [1]. Constipation affects approximately 60–80% of PD patients [2,3]. Currently, the pathogenesis of PD has been postulated as originating in either olfactory or gastrointestinal nerve terminals, with misfolding and aggregation of α-synuclein (α-syn) playing a central role [4]. Therefore, the “dual-hit hypothesis” was formed, which states that the initial formation of α-syn aggregates occurs in the terminal end fields of neurons residing in the olfactory bulb and the parasympathetic neurons of the intestinal plexus to the dorsal motor nucleus of the vagus (DMV) [5,6]. However, it is still unclear whether the cognitive impairment is associated with the gastrointestinal route (caudal to rostral) or is related to the short distance spreading from the olfactory pathway to the neocortex [4,7–10]. It has been shown that compromise of olfactory limbic structures, such as the amygdala, the entorhinal, and the piriform cortices, plays a major role in developing cognitive dysfunction and dementia [11–14]. The olfactory bulb is just one synapse away from these limbic structures [15–17]. It might be conceivable that α-syn pathology gains access to the neocortex by spreading from the olfactory bulb rather than the caudo-rostral route. Several studies have investigated olfaction and constipation in PD, but the exact relationship between hyposmia, constipation, and cognitive impairment has not been explored. In our study, we aimed to elucidate the association of anosmia and constipation with cognitive dysfunction and disease severity in patients with Parkinson’s disease (PD).

## Methods

### Data source and study population

Consecutive and newly diagnosed PD patients (clinical course less than 5 years) were recruited from the neurology outpatient clinic at Taichung Veterans General Hospital from February 2017 to August 2018. The exclusion criteria included the patients who had been diagnosed with dementia before enrollment, atypical parkinsonism diagnosed during the outpatient clinic follow-up, and those who could not cooperate with the tests required in this study. All participants were given a full explanation, in lay terms, of the aims of the study, and provided written informed consent prior to enrollment. The study protocol, subject information leaflet and informed consent document were reviewed and approved by an Institutional Review Board (IRB) at Taichung Veterans General Hospital (No.CE16171B). All methods were performed in accordance with the Declaration of Helsinki and regulations.

### Ascertainment of constipation

We defined participants with constipation according to Rome IV diagnostic criteria. For evaluating the severity of constipation, we collected information on prescribed drug types, dates of prescriptions, duration of the supplement, and a total number of tablets as derived from the pharmacy prescription database. Subjects with constipation were classified as normal (grade 0: no constipation), mild (grade 1: had constipation without medications or using one kind of laxative), moderate (grade 2: using two kinds of laxatives), or severe (grade 3: using more than three kinds of laxatives) [18].

### Ascertainment of anosmia

All the participants finished the Traditional Chinese version of the University of Pennsylvania Smell Identification Test (UPSIT-TC). There are 40 odorants covered with “scratch and sniff” labels in the test, and the participants were asked to choose the correct answer from four choices. According to the previous studies, total anosmia was defined as a UPSIT score less than 19 [19,20].

### Ascertainment of cognitive impairment

The cognitive functions of the participants were evaluated by the Montreal Cognitive Assessment (MoCA). The cognitive impairment group was defined as MoCA less than 26.

### Disease severity of PD

The Movement Disorder Society-Sponsored Revision of the Unified Parkinson’s Disease Rating Scale (MDS-UPDRS) was used for assessment of PD symptoms. There were four parts in the MDS-UPDRS scale to evaluate various aspects of PD including non-motor and motor experiences of daily living and motor complications. The total score (UPDRS T), and the part 3 score (UPDRS 3) representing the severity of motor symptoms were used for further analysis.

### Statistical analysis

All analyses were performed using SPSS (IBM Corporation, Armonk, New York, USA). The student’s *t*-test and chi-squared test were used to compare the age, gender, severity of constipation, MoCA, UPDRS T, and UPDRS 3 between the anosmia group and non-anosmia group. Linear regression was used to demonstrate the relationship between MoCA and UPSIT scores. Binary logistic regression for cognitive impairment was established with covariates, such as age, gender, anosmia, and constipation grades. Multiple linear regression for MDS-UPDRS scores was also performed using age, gender, anosmia, and constipation grades as covariates. All statistical tests were two-sided, conducted at a significance level of 0.05, and reported using *p*-value and 95% confidence intervals (CIs).

## Results

A total of 107 patients were included in this study (Table 1). The MoCA score was significantly lower in the group of total anosmia (22.9 ± 5.9 vs.27.0 ± 2.6, *p*-value < 0.001). With regard to the severity of constipation, there was a difference between the anosmia group and non-anosmia (*p* = 0.02). Post-hoc analysis was performed. The anosmia group had a significantly higher proportion of subjects with severe constipation (grade 3 constipation) (36.8% vs 12.0%, *p* = 0.02). No significant differences in age, sex, UPDRS T, and UPDRS 3 were observed between the two groups.

**Table 1:** Demographic data.

Variables	Non-anosmia (n = 50)	Total anosmia (n = 57)	*P*
Age (Year)	60.9±9.1	67.2±7.9	0.43
Sex (Male), %	33 (66.0)	35 (61.4)	0.68
MoCA	27.0±2.6	22.9±5.9	<0.001
MDS-UPDRS score			
Part 3, motor symptoms	23.8±12.7	33.4±16.5	0.10
Total	40.8±18.5	53.7±26.4	0.06
Constipation grade, %			
0	17 (34.0)	14 (24.6)	0.02
1	17 (34.0)	12 (21.1)	
2	10 (20.0)	10 (17.5)	
3†	6 (12.0)	21 (36.8)	

Total anosmia: Defined as UPSIT score < 19; non-anosmia: Defined as UPSIT score ≥ 19.

MoCA: Montreal cognitive assessment; MDS-UPDRS: Movement disorder society-sponsored revision of the unified Parkinson’s disease rating scale; UPSIT: University of Pennsylvania smell identification test.

† The adjusted standardized residual was greater than 2 which indicated that the column proportions were significantly different at *p* < 0.05 level.

**Fig 1:**
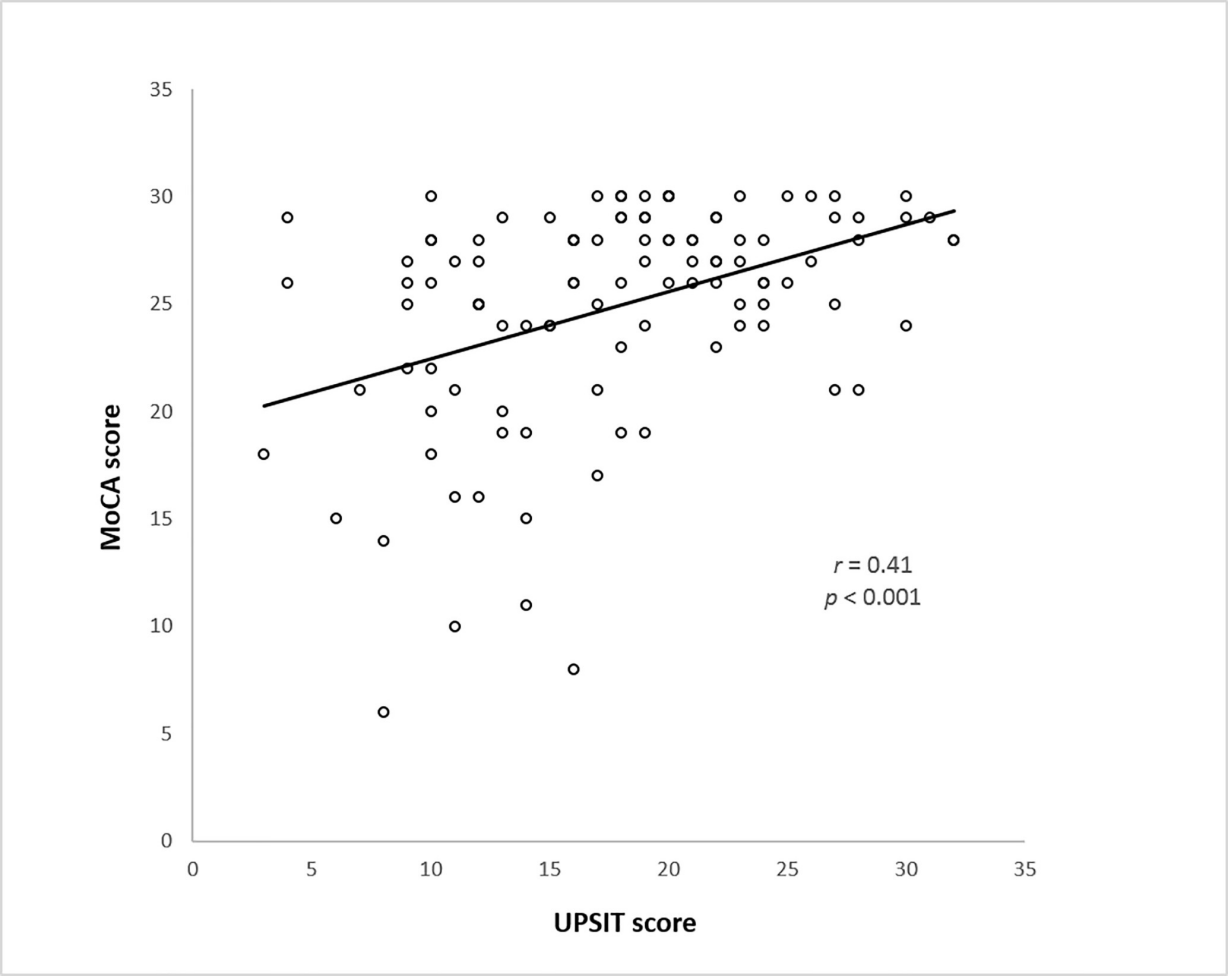
Correlation between MoCA and UPSIT score. *r* = correlation coefficient; MoCA: Montreal cognitive assessment; UPSIT: University of Pennsylvania smell identification test.

Fig 1 revealed the linear regression between MoCA score and UPSIT score with a correlation coefficient of 0.41 (*p* < 0.001). Table 2 shows the odds ratio of covariates associated with cognitive dysfunction (MoCA < 26). The anosmia (odds ratio = 2.63, *p* = 0.03) and aging (odds ratio = 1.10, *p* < 0.001) both showed significant association with cognitive dysfunction.

**Table 2:** The odds ratio of variables associated with cognitive dysfunction (MoCA < 26).

Variable	OR	95% CI of OR	*P*
Anosmia	2.63	1.05–6.55	0.03
Sex (Female)	1.15	0.46–2.88	0.76
Age	1.10	1.04–1.17	<0.001
Constipation grade†			
1	0.85	0.26–2.79	0.79
2	0.55	0.15–2.04	0.37
3	0.69	0.20–2.37	0.56

MoCA: Montreal cognitive assessment; OR: Odds ratio; CI: Confidence interval.

† Reference category: Normal.

Tables 3 and 4 present multiple linear regression analysis results on UPDRS 3 score and UPDRS T score, respectively. Total anosmia was significantly correlated with UPDRS 3 score, with a *β*-coefficient of 7.3 (95% CI = 0.93–13.6, *p* = 0.02). On the contrary, severe constipation was significantly associated with UPDRS T score with *β*-coefficient 14.88 (95% CI = 2.41–27.35, *p* = 0.02).

**Table 3:** Multiple linear regression analysis on MDS-UPDRS part 3 score.

Variable	*B*	95% CI of *B*	*P*
Age	0.08	-0.26–0.42	0.63
Sex (Female)	-0.51	-6.47–5.44	0.86
Anosmia	7.30	0.93–13.67	0.02
Constipation grade†			
1	-4.99	-12.71–2.72	0.20
2	3.74	-4.83–12.31	0.38
3	4.57	-3.66–12.81	0.27

MDS-UPDRS: Movement disorder society-sponsored revision of the unified Parkinson’s disease rating scale; CI: Confidence interval.

*B* = unstandardized regression coefficient.

† Reference category: Normal.

**Table 4:** Multiple linear regression analysis on MDS-UPDRS total score.

Variable	*B*	95% CI of *B*	*P*
Age	0.09	-0.43–0.61	0.72
Sex (Female)	4.31	-4.70–13.34	0.34
Anosmia	7.66	-1.97–17.30	0.11
Constipation grade†			
1	-5.09	-16.77–6.59	0.39
2	7.33	-5.65–20.31	0.26
3	14.88	2.41–27.35	0.02

MDS-UPDRS: Movement disorder society-sponsored revision of the unified Parkinson’s disease rating scale; CI: Confidence interval.

*B* = unstandardized regression coefficient.

† Reference category: Normal.

## Discussion

Two main findings were demonstrated in the current study. First, total anosmia was a factor strongly related to cognitive dysfunction in PD. Second, the severity of constipation was not associated with cognition but was associated with the disease severity of PD. These two findings support the dual-hit hypothesis that olfactory plus gastrointestinal dysfunction is a potential explanation for PD pathogenesis. Accumulating evidence from animal studies indicates the involvement of prion-like mechanisms in the propagation of α-syn pathology [21]. It has been proposed that the neurodegenerative process is initiated via the olfactory bulb or enteric nervous system and, after that, through to the brainstem areas, limbic system, or neocortex. A range of studies have suggested that brain regions related to olfactory function are closely associated with cognitive decline and also correspond to our finding that anosmia is a prominent clinical feature that predicts the subsequent development of PD dementia (PDD) [22–26]. Also, this study suggested that, in the cognitive phenotype of PD, the transmission of α-syn may be initiated from the olfactory structures rather than from the enteric nervous system with caudo-rostral propagation owing to the fact that, in this study, constipation was not associated with cognitive impairment in newly diagnosed PD patients, but anosmia was.

Although vagal and olfactory routes seem to be independent, a growing body of evidence suggests that these pathways may be interconnected at several levels, including bidirectional spreading. Among the olfactory structures, the anterior olfactory nucleus, a nodal point in the olfactory system, is an early and preferential site of α-syn pathology connected with the remainder of the olfactory structures and also with numerous ‘non-olfactory’ structures [21]. Retrograde vagal pathways potentially reach the anterior olfactory nucleus. This might explain our finding that only those PD patients with severe constipation demonstrated an increased prevalence of total anosmia.

In our study, multiple linear regression analysis showed significant correlations between total anosmia and UPDRS 3 score. In a previous cross-sectional study, it has been observed that abnormal odor identification was associated with advanced stages of PD and increasing severity of motor symptoms, even though disease duration was not a significant predictor [27]. Our findings are consistent with these prior results. In contrast, severe constipation was more correlated with UPDRS T score than with UPDRS 3 score. This result may be explained by the fact that UPDRS T contains non-motor symptoms, such as gastrointestinal tract symptoms and sleep problems. These non-motor symptoms possibly resulted from synucleinopathy at the brainstem where the gut-brain axis and Braak staging system meet.

In conclusion, here we found that anosmia but not constipation was associated with cognitive impairment in PD patients. Nevertheless, severe constipation was associated with PD disease severity. We suggest that the propagation of α-syn from the olfactory route is distinct from the enteric nervous system, but the intercommunication between these two routes is complex. Since olfactory dysfunction is a plausible major predictor of the subsequent development of PDD, the UPSIT olfactory test might be recommended to be included in the routine clinical assessment of PD to early detection of dementia occurrence.

## Supporting information

S1 File (XLSX)
